# Comparative analysis of *KNOX* genes and their expression patterns under various treatments in *Dendrobium huoshanense*

**DOI:** 10.3389/fpls.2023.1258533

**Published:** 2023-10-04

**Authors:** Guohui Li, Muhammad Aamir Manzoor, Guoyu Wang, Cunwu Chen, Cheng Song

**Affiliations:** ^1^Anhui Engineering Research Center for Eco-agriculture of Traditional Chinese Medicine, Anhui Dabieshan Academy of Traditional Chinese Medicine, College of Biological and Pharmaceutical Engineering, West Anhui University, Lu’an, China; ^2^Department of Plant Science, School of Agriculture and Biology, Shanghai Jiao Tong University, Shanghai, China; ^3^College of pharmacy, Anhui University of Chinese Medicine, Hefei, China

**Keywords:** KNOX, abiotic stress, gene expression, *D. huoshanense*, genome

## Abstract

**Introduction:**

KNOX plays a pivotal role in governing plant growth, development, and responses to diverse abiotic and biotic stresses. However, information on the relationship between the *KNOX* gene family and expression levels under different treatments in *Dendrobium* is still limited.

**Methods:**

To address this problem, we first used bioinformatics methods and revealed the presence of 19 *KNOX* genes distributed among 13 chromosomes in the *Dendrobium huoshanense* genome. Through an analysis of phylogenetic relationships, these genes were classified into three distinct clades: class I, class II, and class M. Our investigation included promoter analysis, revealing various *cis*-acting elements associated with hormones, growth and development, and abiotic stress responses. Additionally, qRT-PCR experiments were conducted to assess the expression patterns of *DhKNOX* genes under different treatments, including ABA, MeJA, SA, and drought.

**Results:**

The results demonstrated differential expression of *DhKNOX* genes in response to these treatments, thereby highlighting their potential roles in stress adaptation.

**Discussion:**

Overall, our results contribute important insights for further investigations into the functional characterization of the *Dendrobium KNOX* gene family, shedding light on their roles in plant development and stress responses.

## Introduction

The KNOTTED1-like homeobox (KNOX) is a big transcription factor family that plays a key role in the regulation of plant growth and development (Hamant and Pautot, 2010). KNOX proteins generally have four conserved domains, which are as follows: KNOX1, KNOX2, ELK, and HOX (Gao et al., 2015). The *KNOX* gene family is ubiquitous, from lower plant algae and bryophytes to higher seed plants (Gao et al., 2015). Based on the structural characteristics, expression mode, and phylogenetic relationship of the *KNOX* gene, researchers usually divide it into two subfamilies: class I and class II (Furumizu et al., 2015). Different types of *KNOX* genes exhibit distinct expression patterns and biological functions. Class I KNOX proteins can promote cell proliferation, and their members have a significant role in the formation and maintenance of the plant stem tip meristem (SAM) in angiosperms. In addition, the class I *KNOX* gene is expressed in the apical meristem, while its expression level decreases in lateral buds (axillary buds), and mutants cannot maintain apical meristem (Gao et al., 2015). Moreover, the class I *KNOX* gene is also involved in other development processes, such as compound leaf formation, internode elongation, inflorescence structure, and the establishment of vascular tissue connections between parasitic plants and their hosts (Alakonya et al., 2012; Cheng et al., 2019). Therefore, the regulatory function of the class I *KNOX* gene has been involved in various developmental processes during the development of the stem system.

In higher plants, class II *KNOX* genes have relatively broad tissue specificity, while the expression of class I *KNOX* genes is more limited. Class II *KNOX* gene is expressed in differentiated organs, including leaves, stems, flowers, and roots; its involvement in some developmental processes not only in vascular tissue but also in seed coat development and inflorescence stem in plants (Gao et al., 2015). In *Arabidopsis*, class II genes, such as *KNAT3*, *KNAT4*, and *KNAT5* play a role in lateral organ differentiation due to functional redundancy. The class II *KNOX* family has relatively few studies due to the lack of phenotypes of known mutants. Among class II *KNOX* genes, *KNAT7* has received the most attention and is known to play a role in the transcriptional network regulating secondary cell wall biosynthesis (Furumizu et al., 2015; Gao et al., 2015). However, there are relatively few reports on the function of the class II *KNOX* gene in plants, which is mainly related to the formation of secondary cell walls. For example, the synergistic effect of *KNAT7* and *KNAT3* affects the deposition of secondary cell walls in plants, thereby altering the mechanical support strength of *Arabidopsis* stems (Wang S. M. et al., 2020); class KNATM is a special *KNOX* gene subfamily of dicotyledons. Research on the *KNATM* gene of *Arabidopsis* showed that this gene is involved in regulating leaf polarity and leaf shape (Magnani and Hake, 2008).

Other studies have shown that KNOX is involved in a variety of developmental processes, mainly by influencing hormone metabolism and signal transduction pathways (Bolduc and Hake, 2009). KNOX activates the biosynthesis of plant cytokinin (Yanai et al., 2005). For instance, MtKNOX3 activates the biological isopentenyl transferase (IPT) gene of cytokinin in apples, regulates the development of nodules, and activates the biosynthesis of cytokinin during nodulation (Azarakhsh et al., 2015). KNOX protein can inhibit the production of gibberellin (GA), and KNOX negatively regulates the accumulation of GA by controlling the abundance of GA2 oxidase (Bolduc and Hake, 2009). In addition, the KNOX protein also participates in other hormone signal transduction pathways. KNOX changes the abundance of proteins related to auxin transporter signal transduction components, thus regulating tomato abscission (Ma et al., 2015). During the germination and early seedling development of *Arabidopsis*, KNAT3 interacts with BELL-like homeodomain (BLH) proteins and cooperatively regulates the ABA response (Dachan et al., 2013).

*Dendrobium huoshanense* is a perennial epiphytic herb belonging to the Orchidaceae family, and it holds the status of a nationally protected variety of traditional Chinese medicine. This plant species possesses significant medicinal value (Liu et al., 2018; Wang et al., 2022), which is considered the best all *Dendrobium* species due to its significant immune regulation, anti-inflammatory, and anticancer activities (Lin et al., 2014; Zha et al., 2017). In addition, clinical studies have found that polysaccharides from *Dendrobium huoshanense* have a significant inhibitory effect on the apoptosis of human lens epithelial cells (Zha et al., 2017). The continuous improvement of the genome information of *D. huoshanense* (Han et al., 2020) will provide an opportunity to analyze the *KNOX* gene, which plays significant roles in plant growth and development, as well as the response of *Dendrobium* to different environmental stress conditions. Although KNOX proteins have been reported in *Arabidopsis* and maize, there is limited research on the identification and expression analysis of *KNO*X gene family members in *Dendrobium*. In this study, a bioinformatics approach was employed to identify the *KNOX* gene in *Dendrobium*. The analysis combined the characteristics of the *KNOX* gene family as well as the basic physical and chemical properties, chromosome distribution, evolutionary relationship, gene structure, *cis*-acting elements, and expression patterns under different stress conditions. These findings lay the foundation for further investigation into the function and regulatory mechanisms of the *KNOX* gene in *Dendrobium* plants.

## Materials and methods

### Plant materials and growth conditions

Tissue-cultured seedlings of *D. huoshanense* were subjected to a sterilization process and then planted on Murashige and Skoog (MS) media. This cultivation occurred in the tissue culture room with long-day conditions (16 h light, 20°C/8 h dark) of West Anhui University’s Anhui Engineering Technology Research Center of Plant Cell Engineering, located in Lu’an City, Anhui Province. The methodology employed in this study was based on previously published articles (Cao et al., 2019). Subsequently, 100 μM MeJA (Aladdin, Shanghai of China), 100 μM SA (Aladdin, Shanghai of China), and 100 μM ABA (Aladdin, Shanghai of China) were added after 0.22 μM microporous filtration. Referring to the method of Cao et al. (2015), 20% PEG6000 was added to the culture medium to simulate drought stress. The leaves were sampled at 1 h, 4 h, and 16 h after treatment. There were three biological replicates per set of samples, and each sample was collected individually for each induction treatment and immediately stored at −80°C to facilitate RNA isolation. In addition, untreated *D. huoshanense* leaves were used as the control group for comparative purposes.

### Identification of *D. huoshanense KNOX* gene family

Using the BLAST tool available on the *Dendrobium* genome (https://ftp.cngb.org/pub/CNSA/data3) information website, a comparative analysis was conducted based on the KNOX protein sequence in *Arabidopsis* as the reference sequence (Furumizu et al., 2015), with a BLASTp (*E*-value = 0.001) and other default parameters. The objective was to identify candidate *KNOX* genes within the *Dendrobium* genome. Furthermore, the conserved KNOX1 (PF03790) or KNOX2 (PF03791) domains should be selected for further analysis using the structural properties that were identified using the Pfam website (Cheng et al., 2019). Subsequently, the isoelectric point and molecular weight of the *KNOX* gene family members of *D. huoshanense* were analyzed by ProtParam (https://web.expasy.org/protoparam/) (Finn et al., 2013). We predict of subcellular localization of the KNOX protein on the website WoLF PSORT (https://wolfpsort.hgc.jp/).

### Construction of the phylogenetic tree and analysis of gene structures

To elucidate the evolutionary relationship of the *DhKNOX* gene, we employed MEGA 5.2 software to construct a *KNOX* gene phylogenetic tree with 1,000 bootstrap values (Tamura et al., 2011). The GenBank accession codes were used to construct the phylogenetic tree (**Supplementary Table S1**). According to the original annotation file in GFF3 format, the CDS of *DhKNOXs* was used for gene structure analysis and visualized with TBtools (Chen et al., 2018).

### Chromosome mapping of *DhKNOX* gene family members

The chromosome location information of the *KNOX* gene family members was obtained from the *Dendrobium* genome annotation file. The *KNOX* gene information from the *Dendrobium* genome database was utilized to perform chromosome localization using the MapDraw V2.1 software (Lee et al., 2013).

### Analysis of *cis*-acting elements of *DhKNOX* gene

TBtools software was used to obtain the 2,000-bp promoter region upstream of the CDS sequence of each *KNOX* gene *Dendrobium* from the genome database and to analyze *cis*-acting elements in the *KNOX* gene promoter. The obtained promoter region sequence was submitted to the PlantCARE database to predict *cis*-acting elements (Cheng et al., 2019). We implement the prediction of protein interactions among members of the *DhKNOX* gene family through the STRING website (https://cn.string-db.org) (Yang et al., 2022).

### Identification of conserved motifs

The sequence alignment of KNOX proteins was performed using ClustalX software (Cao et al., 2016). Additionally, the conserved motifs of KNOX proteins were identified by the MEME online tools (Bailey et al., 2015).

### RNA isolation and qRT-PCR analysis

The total RNA from *D. huoshanense* leaves was extracted using a plant RNA extraction kit (Tiangen). qRT-PCR was used to detect the expression level of the *KNOX* gene at different times after treatment. The internal control gene was used as a reference (Fan et al., 2016). The specific primers for each gene are shown in **Supplementary Table S2**. The qRT-PCR reaction system was 20 µL, and the reaction procedure was 95°C for 3 min, 95°C for 10 s, 60°C for 30 s, and 45 cycles. The melting (65°C~95°C) program is finally run after signal acquisition. Three biological repeats were set for each sample, and each biological duplicate sample was tested three times. Finally, the relative gene expression of *KNOX* genes was calculated using 2^−ΔΔCt^ methods (Livak and Schmittgen, 2001).

## Results

### Identification and characterization of *Dendrobium KNOX* family members

The query sequence used for this analysis consisted of the KNOX family members from *Arabidopsis thaliana*, totaling nine sequences. Based on this query, 19 nonredundant KNOX family members were identified from the genome of *Dendrobium*. These newly identified members were named DhKNOX1 to DhKNOX19. **Table 1** presents various characteristics of the *DhKNOX* genes in *Dendrobium*. The coding region lengths of the *DhKNOX* genes range from 369 to 1,872 bp, while the corresponding coding amino acid lengths range from 123 to 624. The molecular weight of the DhKNOX proteins spans from 18.81 to 69.0 kD, and their isoelectric points range from 4.29 to 10.25. Through the examination of predicted subcellular localization, it was ascertained that the nucleus emerges as the predominant site for the subcellular localization of the majority of *DhKNOX* genes. This observation is in consonance with their established functions as transcription factors.

**Table 1:** Sequence characteristics of 19 *KNOX*s identified in *D. huoshanense*.

Classes	Gene name	Gene code	Length (bp)	Chr	Length (aa)	MW (kDa)	p*I*	Subcellular localization
CLASSI	*DhKNOX1*	Dhu000022539	906	Chr5	302	33.6	5.85	Nucl
	*DhKNOX2*	Dhu000026725	450	Chr8	150	18.1	10.25	Nucl
	*DhKNOX3*	Dhu000012806	492	Chr13	161	18.9	7.07	Nucl
	*DhKNOX4*	Dhu000000755	399	Chr16	133	15.38	7.19	Nucl
CLASSII	*DhKNOX5*	Dhu000002623	450	Chr16	150	17.6	6.21	Nucl
	*DhKNOX7*	Dhu000010068	441	Chr17	147	17.4	5.96	Nucl
	*DhKNOX8*	Dhu000025210	1038	Chr16	346	38.7	9.10	Nucl
	*DhKNOX9*	Dhu000022035	1947	Chr18	649	71.6	6.79	Nucl
	*DhKNOX10*	Dhu000013192	1767	Chr19	589	65.9	6.23	Nucl
	*DhKNOX11*	Dhu000023874	1458	Chr10	486	55.2	6.88	Nucl
	*DhKNOX12*	Dhu000028078	369	Chr16	123	13.2	4.29	Cyto
	*DhKNOX13*	Dhu000028038	1665	Chr16	555	61.3	6.89	Nucl
	*DhKNOX14*	Dhu000012269	1500	Chr17	500	56.6	6.47	Nucl
	*DhKNOX15*	Dhu000027956	1251	Chr4	417	47.8	5.84	Nucl
	*DhKNOX16*	Dhu000025461	1443	Chr7	481	53.6	8.53	Nucl
	*DhKNOX17*	Dhu000004237	1872	Chr3	624	69.0	5.48	Nucl
	*DhKNOX18*	Dhu000010065	843	Chr17	281	30.9	5.35	Nucl
	*DhKNOX19*	Dhu000010069	792	Chr17	264	28.5	5.62	Cyto
CLASSM	*DhKNOX6*	Dhu000012804	795	Chr13	253	27.9	9.02	Nucl
	*DhKNOX12*	Dhu000028078	369	Chr16	123	13.2	4.29	Cyto

### Multiple sequence alignment of DhKNOX proteins

**Figure 1:**
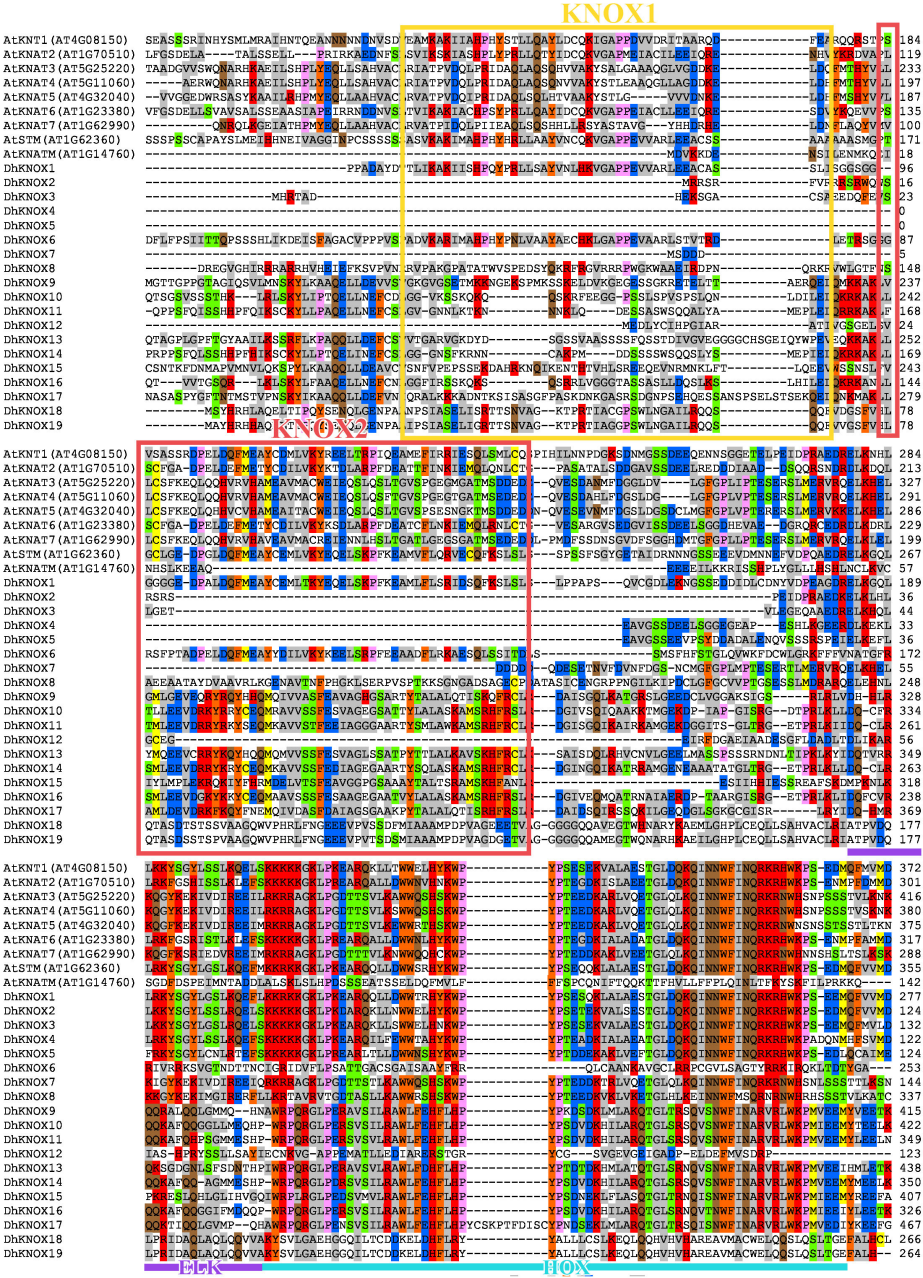
Multiple sequence alignments and domain compositions of DhKNOX proteins.

Multiple sequence alignments revealed that the KNOX protein sequence of *Dendrobium* has four relatively conserved domains (**Figure 1**). The KNOX1 and KNOX2 domains are located at the N-terminus of the protein, while ELK and HOX are located at the C-terminus of the protein. The HOX domain demonstrates the highest level of conservation and exhibits the typical structural characteristics found in the TALE homeobox protein superfamily. In addition, the conserved domains of KNOX1, KNOX2, and ELK exhibit notable conservation. For instance, the conserved domain KNOX2 displays a highly conserved E-L-D amino acid sequence, while the conserved domain ELK features a highly conserved E-L-K amino acid sequence.

### Chromosome mapping of the *KNOX* gene in *Dendrobium*

**Figure 2:**
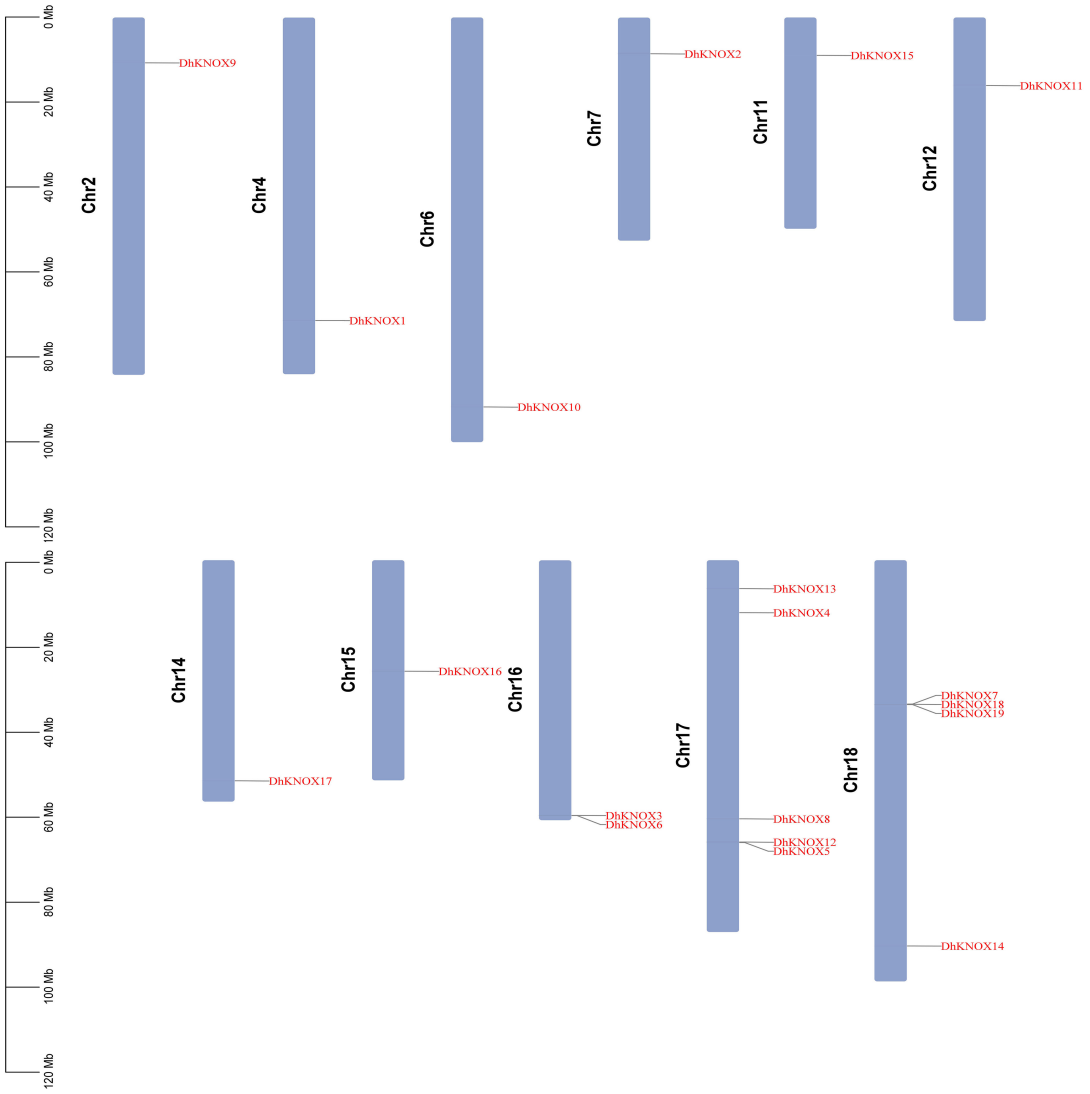
Chromosomal localization of *DhKNOX* genes.

To elucidate the distribution of individual *DhKNOX* members within the *Dendrobium* genome, a distribution map was generated. This map visually depicts the arrangement of the 19 *DhKNOX* genes across each specific chromosome of *Dendrobium*, as shown in **Figure 2**. The 19 *DhKNOX* genes have been clearly located on chromosomes and are distributed on 11 different chromosomes. The distribution of *DhKNOX* genes across the 11 chromosomes of *Dendrobium* is widely scattered. Specifically, chromosomes 16, 17, and 18 harbor two, five, and four *DhKNOX* genes, respectively, while the remaining eight chromosomes each contain one *DhKNOX* gene. Furthermore, a notable observation is that the majority of *DhKNOX* genes are located toward the ends of the chromosomes.

### Phylogenetic analysis of the *DhKNOX* gene

**Figure 3:**
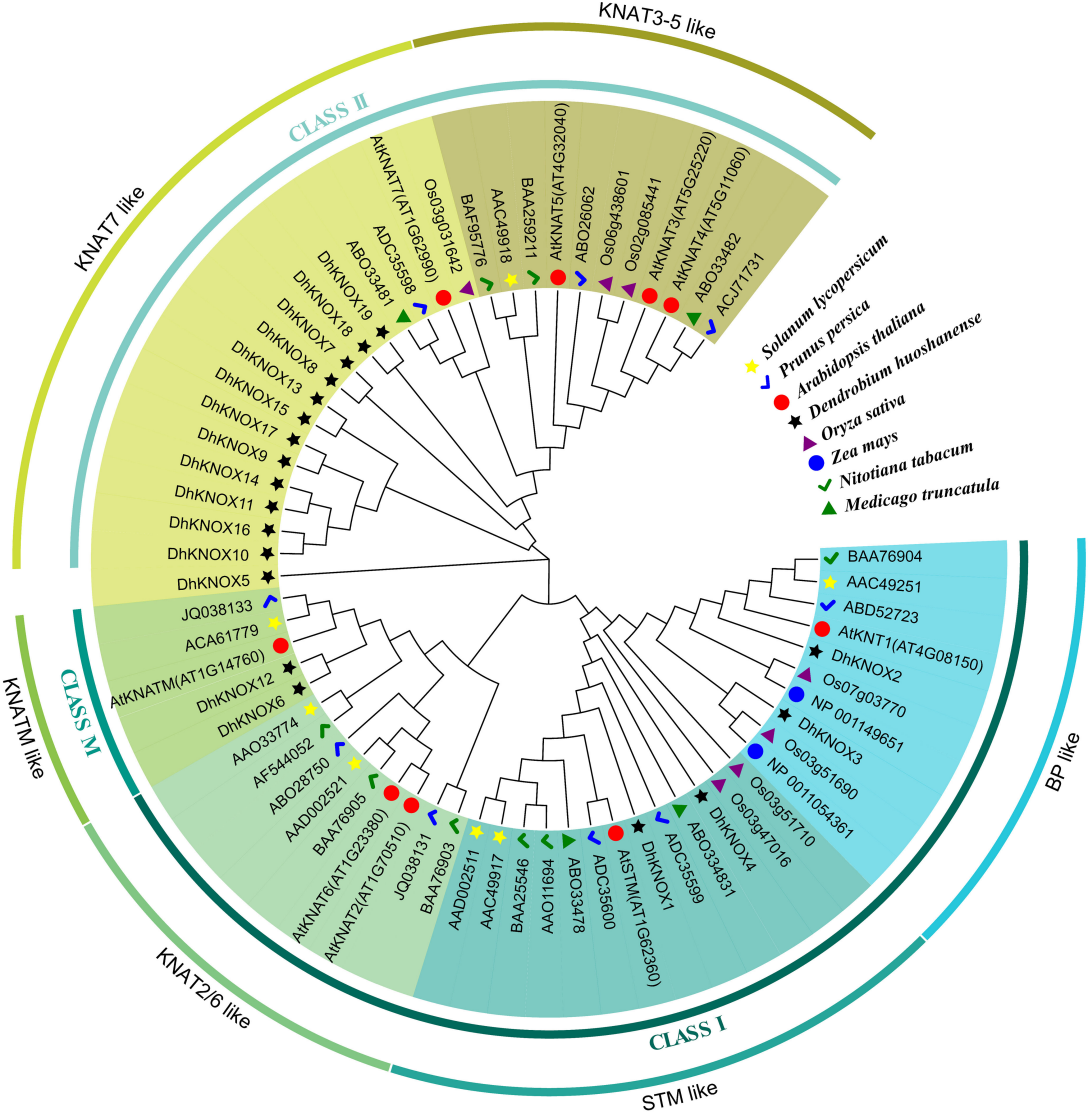
Phylogenetic analysis of the KNOX family in plants.

The KNOX protein sequences of well-known model plants such as *Arabidopsis* and rice, along with the KNOX protein sequence of *D. huoshanense* were constructed using MEGA 5.2 software (**Figure 3**). The results showed that these *KNOX* genes were divided into three categories: class I, class II, and class KNATM. We further analyzed and found that these proteins include six subfamilies: class I includes three subfamilies, class II consists of two subfamilies, and class M contains only the KNATM-like subfamily. In these three large families, most *DhKNOX* genes are distributed in class II. Among the DhKNOX proteins, both the BP-like subfamily and STM-like subfamily contains two DhKNOX members. While there is no member of the KNAT2/6-like subfamily, the other 13 DhKNOX proteins are classified as belonging to the KNAT7-like subfamily. Previous research reported that class I KNOX genes are related to lignin metabolism (Cheng et al., 2019). In our study, *DhKNOX2* and *DhKNOX3* belong to the BP-like subfamily and speculated that they may have similar biological functions. Interestingly, Scofield et al. (2014) demonstrated in *Arabidopsis* that STM-like proteins can regulate the expression level of the *AtKNAT1/BP* gene. *DhKNOX1* and *DhKNOX4* belong to the same subfamily, so we speculate that they may have similar biological functions.

### Analysis of *DhKNOX* gene structure and conserved domain

**Figure 4:**
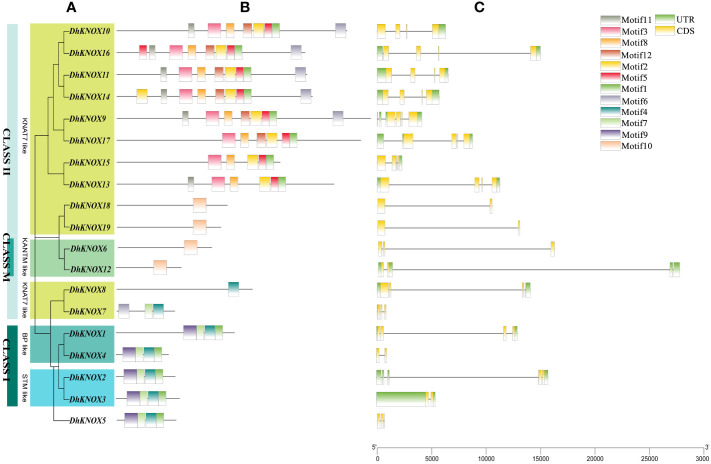
The gene structure and protein-conserved domain of the *DhKNOX* gene family. **(A)** Phylogenetic tree of *DhKNOX* genes. **(B)** Exon-intron structure of *DhKNOX* members. **(C)** Conserved motifs of *DhKNOX* proteins.

To gain deeper insights into the *Dendrobium KNOX* gene family, we utilized the intraspecific phylogenetic tree (**Figure 4A**) and protein-conserved domain distribution (**Figure 4B**), as well as the gene structure of *DhKNOX* genes (**Figure 4C**). Subsequently, employing the MEME website (**Figure 4B**), we identified 10 conserved motifs across the 19 DhKNOX proteins. Significantly, almost all DhKNOX proteins encompass at least one conserved domain, such as motif1, motif3, motif4, and motif10. This observation reinforces the reliability of the *DhKNOX* gene screening results. However, it is worth mentioning that *DhKNOX6* and *DhKNOX12* in class M are exceptions, lacking conserved domains. Furthermore, from an evolutionary perspective, the *DhKNOX* genes within the same subfamily and exhibiting close relationships also demonstrate consistency in gene structure composition and protein domain allocation, which supports their close evolutionary relationships.

The findings indicated that all *DhKNOX* genes comprised both exons and introns, and the *DhKNOX* gene family predominantly exhibited a pattern of four to six exons and three to five introns. The number of gene exons in class I was between three and six, while almost all genes in class II contained five or six exons (**Figure 4C**). Among the DhKNOX family members, *DhKNOX3*, *DhKNOX4*, *DhKNOX18*, and *DhKNOX19* contain only one intron. Most DhKNOX members have three introns. Generally, closely related members have similar genetic structures, such as *DhKNOX11/DhKNOX14* and *DhKNOX15/DhKNOX13*. It is noteworthy that except for *DhKNOX5*, *DhKNOX9*, and *DhKNOX15*, class I genes contain a long intron (**Figure 4C**), which is consistent with the structural characteristics of the class I KNOX gene family (Morimoto et al., 2005).

### Analysis of *cis*-acting elements in *DhKNOX* gene gamily promoters

**Figure 5:**
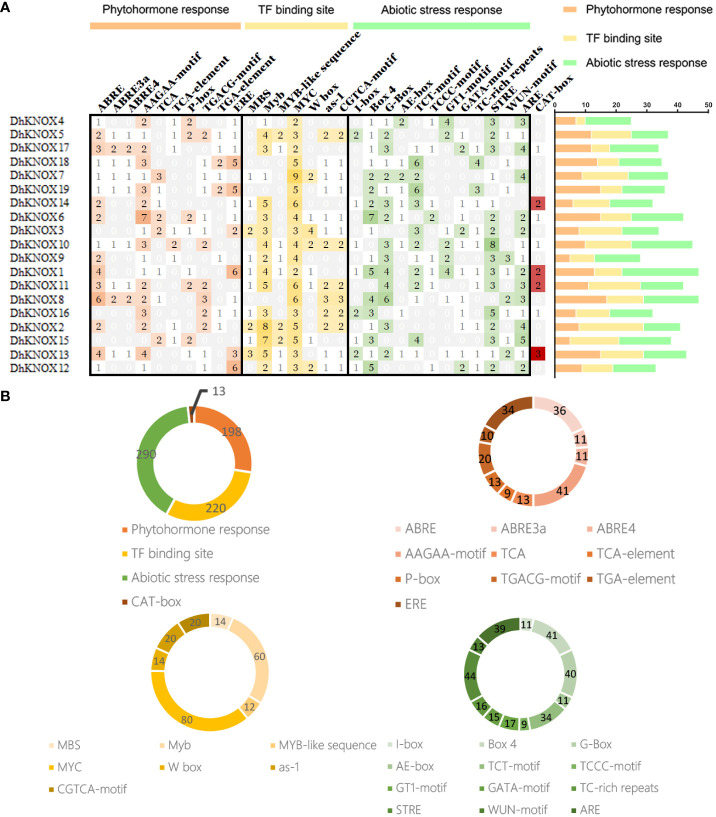
Analysis of cis-acting elements of the *KNOX* genes in *D. huoshanense*. **(A)** The different colors and numbers of the grid indicated the numbers of different promoter elements in these *KNOX* genes. The different colored histogram represented the sum of the *cis*-acting elements in each category. **(B)** Pie charts of different sizes indicated the ratio of each promoter element in each category, respectively.

To analyze the potential expression regulation mechanism of *DhKNOX* gene family members, we identified the *cis*-acting elements of each member (**Figure 5**). For the identified *cis*-acting elements, the elements related to plant hormones, plant growth, participation in stress responses, and light responses were selected for analysis. First, based on the 2,000-bp promoter sequence, stress-related elements, including LTR, MBS, ARE, and other elements, were identified, and transcriptional regulatory-related elements, including MYB, W-box, and MYB-also, were found. In addition, we discovered and validated a couple of elements related to plant hormones, such as P-box, TGA-element, ABRE, and other elements. It is worth noting that the most common motif is the *cis*-acting element of the motif related to ABA reactivity, accounting for 36% of the hormone response motifs scanned. *Cis*-acting elements of the TGACG motif related to MeJA reactivity accounted for 13%. In addition, we found that the TCA element in response to SA, among the 19 *DhKNOX* gene promoters, appeared 29 times and accounted for 10%. The MBS element was related to drought stress, which accounted for 12%. These findings suggest that the transcription of *DhKNOX* genes may be affected by these hormones and drought stress.

### Prediction of protein interactions of *DhKNOX* gene family members

**Figure 6:**
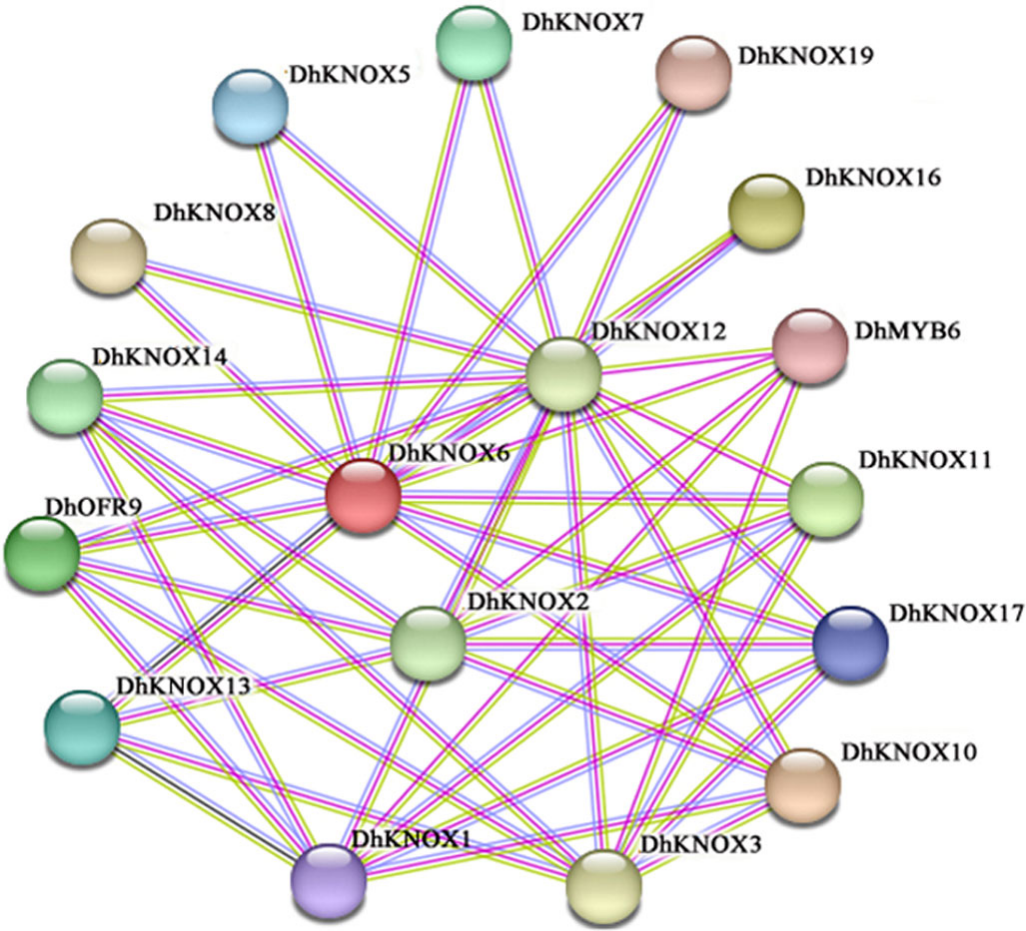
Prediction of protein interactions within *D. huoshanense KNOX* gene family.

We conducted protein interaction predictions among the members of the *DhKNOX* gene family by using the STRING website (**Figure 6**). The results revealed a significant number of interactions among the family members. We found protein-to-protein interactions among 15 members of the DhKNOX family, including DhKNOX1, DhKNOX2, DhKNOX3, DhKNOX5, and DhKNOX19. There are 17 nodes and 42 sets of interaction relationships in the interaction network. DhKNOX2, DhKNOX6, and DhKNOX12 are located at the center of the entire protein interaction network and can interact with the proteins DhOFP9 and DhMYB6. In summary, the members of the *DhKNOX* gene family have the ability to form dimers or polymers through protein interactions and to carry out transcriptional regulatory functions.

### Expression analysis of the *DhKNOX* genes under hormonal treatments

Various abiotic stresses may affect the growth and development of plants and ultimately affect the regulation of a series of stress-related genes (Grallath et al., 2005). Consequently, it becomes imperative to clarify the regulatory pathway and identify pivotal regulatory factors implicated in hormone responses in *D. huoshanense*. To better understand the changes in the expression level of the *DhKNOX* gene under various hormone treatments, we performed qRT-PCR experiments to analyze their expression patterns under MeJA, ABA, and SA treatments.

**Figure 7:**
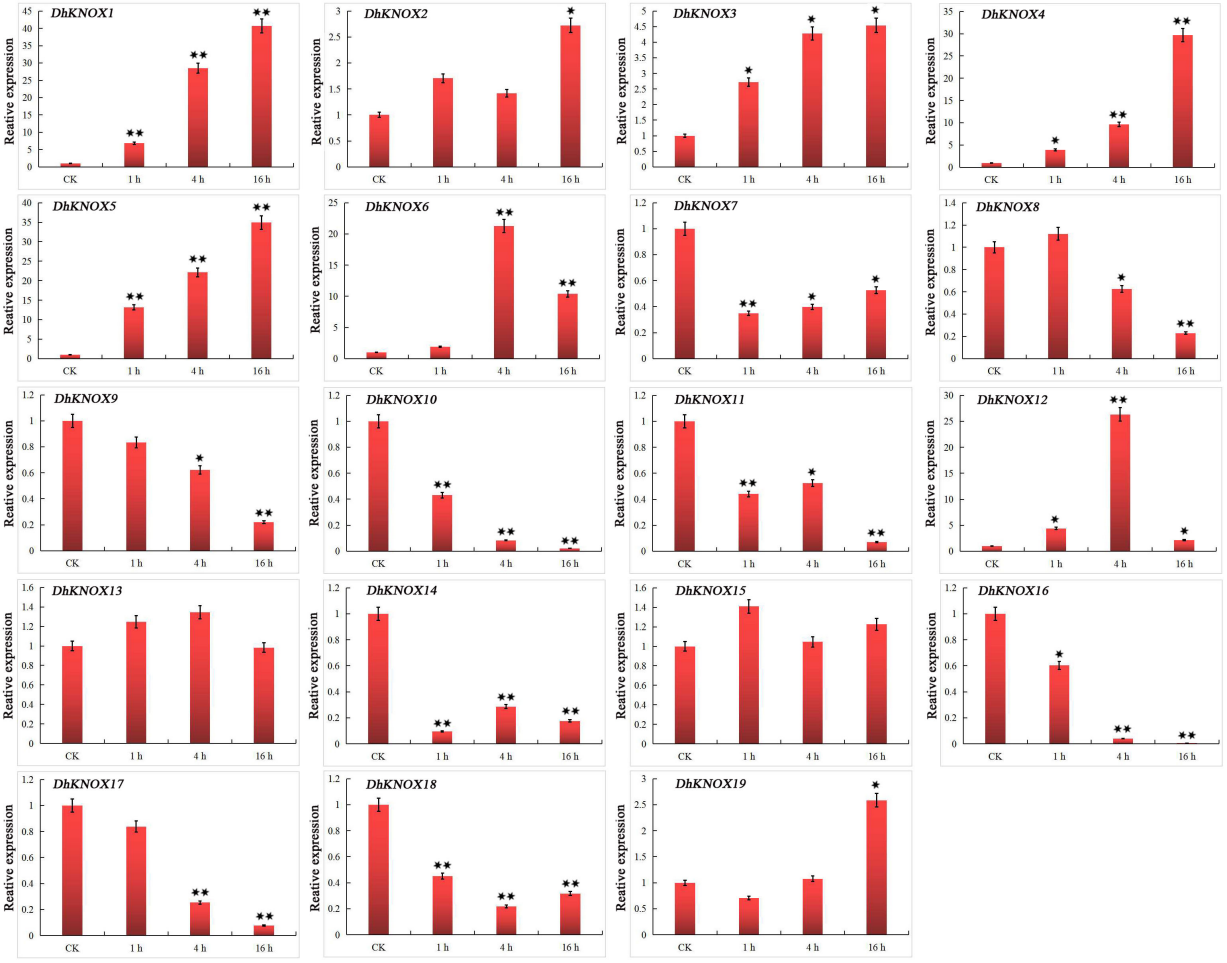
Expression patterns of *DhKNOX* genes in response to MeJA hormone treatments. The *x*-axis indicated the time course of each stress treatment, and the *y*-axis represented the relative expression level. The expression data of each gene at 0 h were used as a control sample to show the relative expression level. The data are presented as the mean values ± standard deviation of three biological replicates. Significant differences were determined by ANOVA combined with a *post-hoc* test (Student’s *t*-test). An asterisk indicates significant differences (^*^p < 0.05; ^**^p < 0.01).

After treatment with MeJA, we found that the expression of 10 *DhKNOX* genes was downregulated to different degrees (**Figure 7**). The results showed that *DhKNOX1*, *DhKNOX2*, *DhKNOX3*, *DhKNOX4*, *DhKNOX5*, *DhKNOX6*, and *DhKNOX12* were obviously significantly and rapidly upregulated at all time points. Among these *DhKNOX* genes, we found that the highest expression levels of *DhKNOX1*, *DhKNOX2, DhKNOX3*, *DhKNOX4*, and *DhKNOX5* occurred 16 h after treatment. The expression of one *DhKNOX6* and *DhKNOX12* gene peaked at 4 h. Notably, it was found that four genes, *DhKNOX1*, *DhKNOX2*, *DhKNOX3*, and *DhKNOX4*, were all clustered in class I, and *DhKNOX6* and *DhKNOX12* were clustered in class II. Moreover, eight *DhKNOX* genes presented reduced expression levels to different degrees. Interestingly, we found that the expression of two *DhKNOX* genes (*DhKNX13* and *DhKNOX15*) was not obviously changed at any time point, and the two *DhKNOX* genes lacked the TGACG motif.

**Figure 8:**
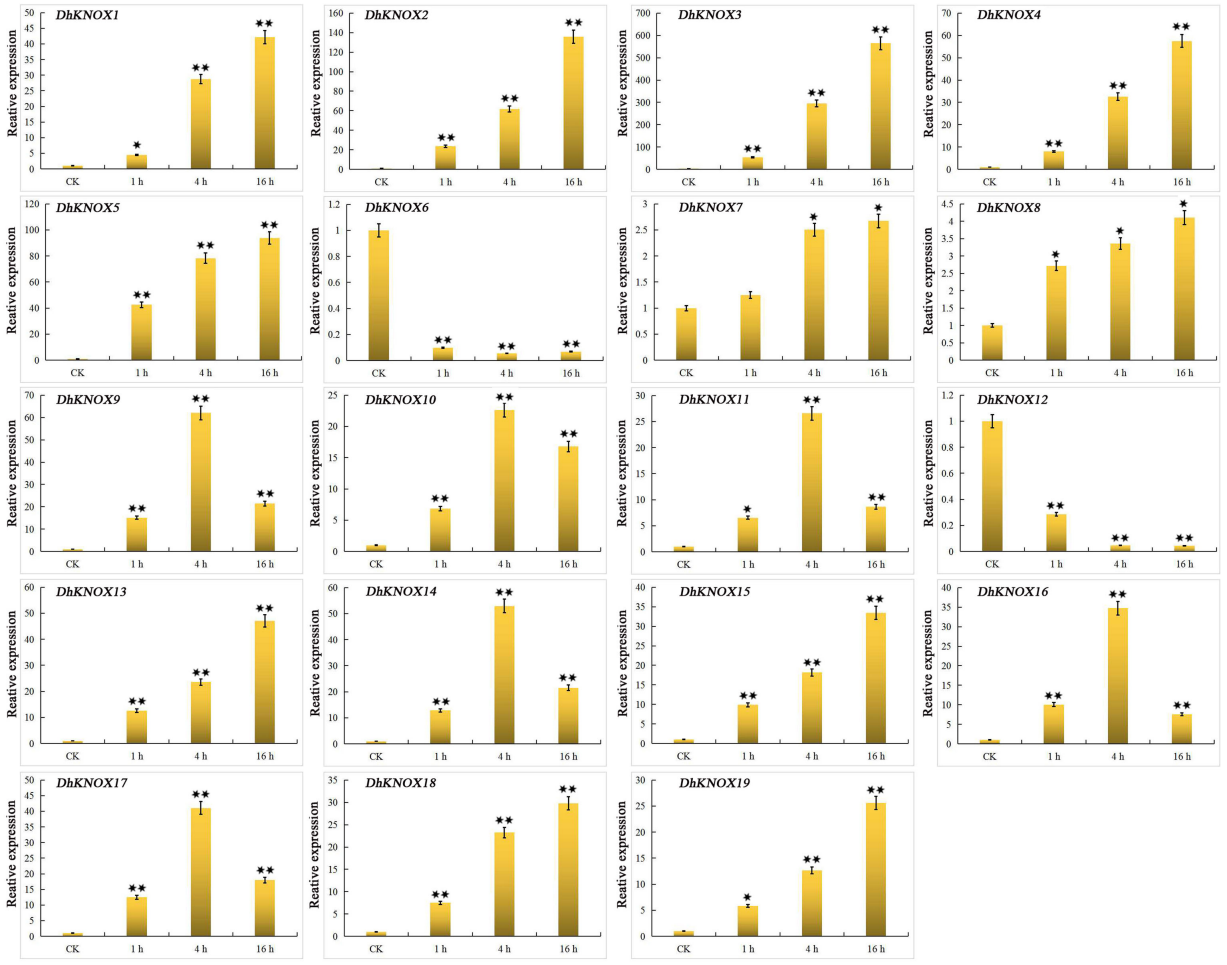
Expression patterns of *DhKNOX* genes in response to ABA hormone treatments. The *x*-axis indicated the time course of each stress treatment, and the *y*-axis represented the relative expression level. The expression data of each gene at 0 h were used as a control sample to show the relative expression level. The data are presented as the mean values ± standard deviation of three biological replicates. Significant differences were determined by ANOVA combined with a *post-hoc* test (Student’s *t*-test). An asterisk indicates significant differences (^*^p < 0.05; ^**^p < 0.01).

ABA induced the transcription of 19 *DhKNOX* genes. The expression levels of 17 *DhKNOX* genes increased to varying degrees (**Figure 8**). Among them, 11 *DhKNOX* genes were significantly upregulated at the last time point (16 h), such as *DhKNOX2* and *DhKNOX3*, which were upregulated by more than 100- and 500-fold, respectively. Interestingly, we found that the expression of two *DhKNOX* genes was clustered in class I. However, we found that *DhKNOX2* had only two ABREs, and *DhKNOX3* lacked the ABREs. In addition, two *DhKNOX* genes (*DhKNOX6* and *DhKNOX12*) clustered in class II, and their expression levels decreased significantly.

**Figure 9:**
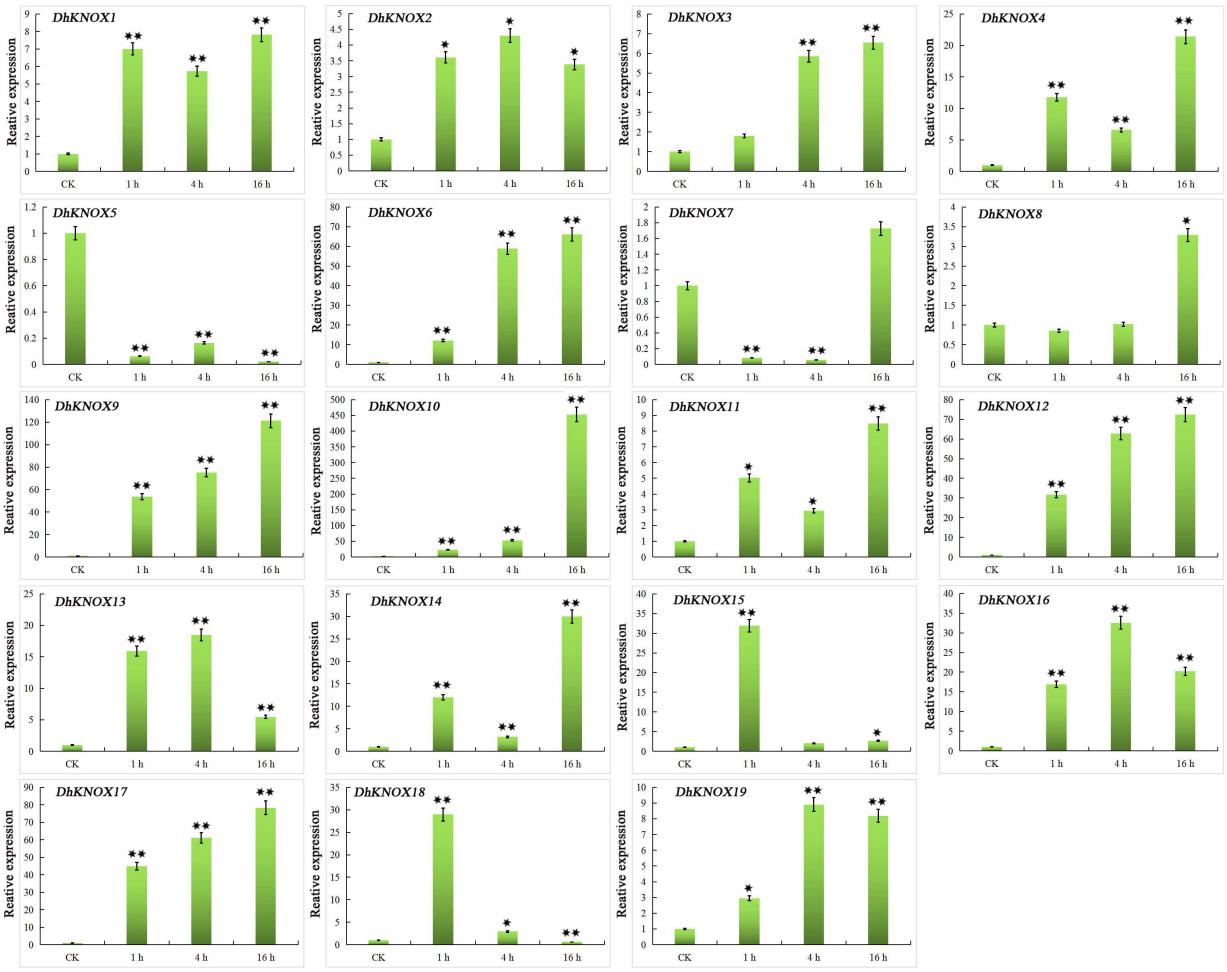
Expression patterns of *DhKNOX* genes in response to SA hormone treatments. The *x*-axis indicated the time course of each stress treatment, and the *y*-axis represented the relative expression level. The expression data of each gene at 0 h were used as a control sample to show the relative expression level. The data are presented as the mean values ± standard deviation of three biological replicates. Significant differences were determined by ANOVA combined with a *post-hoc* test (Student’s *t*-test). An asterisk indicates significant differences (^*^p < 0.05; ^**^p < 0.01).

Under SA treatment, in leaves, the expression level of 17 *DhKNOX* genes increased significantly at three treatment time points (1 h, 4 h, and 16 h) (**Figure 9**). We further found that the highest expression levels of 12 *DhKNOX* genes occurred after 16 h of treatment. The expression pattern of most other *DhKNOX* genes showed a trend of increasing first and then decreasing, and *DhKNOX15* and *DhKNOX18* had the highest expression levels after 1 h. Four of 17 genes reached their highest expression level after 4 h of SA treatment and 4 h but decreased after 16 h, such as *DhKNOX2*, *DhKNOX13*, *DhKNOX16*, and *DhKNOX19*. Of these four *DhKNOX* genes, only *DhKNOX2* was clustered in class I and had one TCA element. Notably, *DhKNOX13*, *DhKNOX16*, and *DhKNOX19* were clustered in class I, and all lacked TCA elements. Only two *DhKNOX* genes were obviously rapidly and significantly downregulated at all time points.

### Expression analysis of the *DhKNOX* gene under PEG-mediated drought conditions

**Figure 10:**
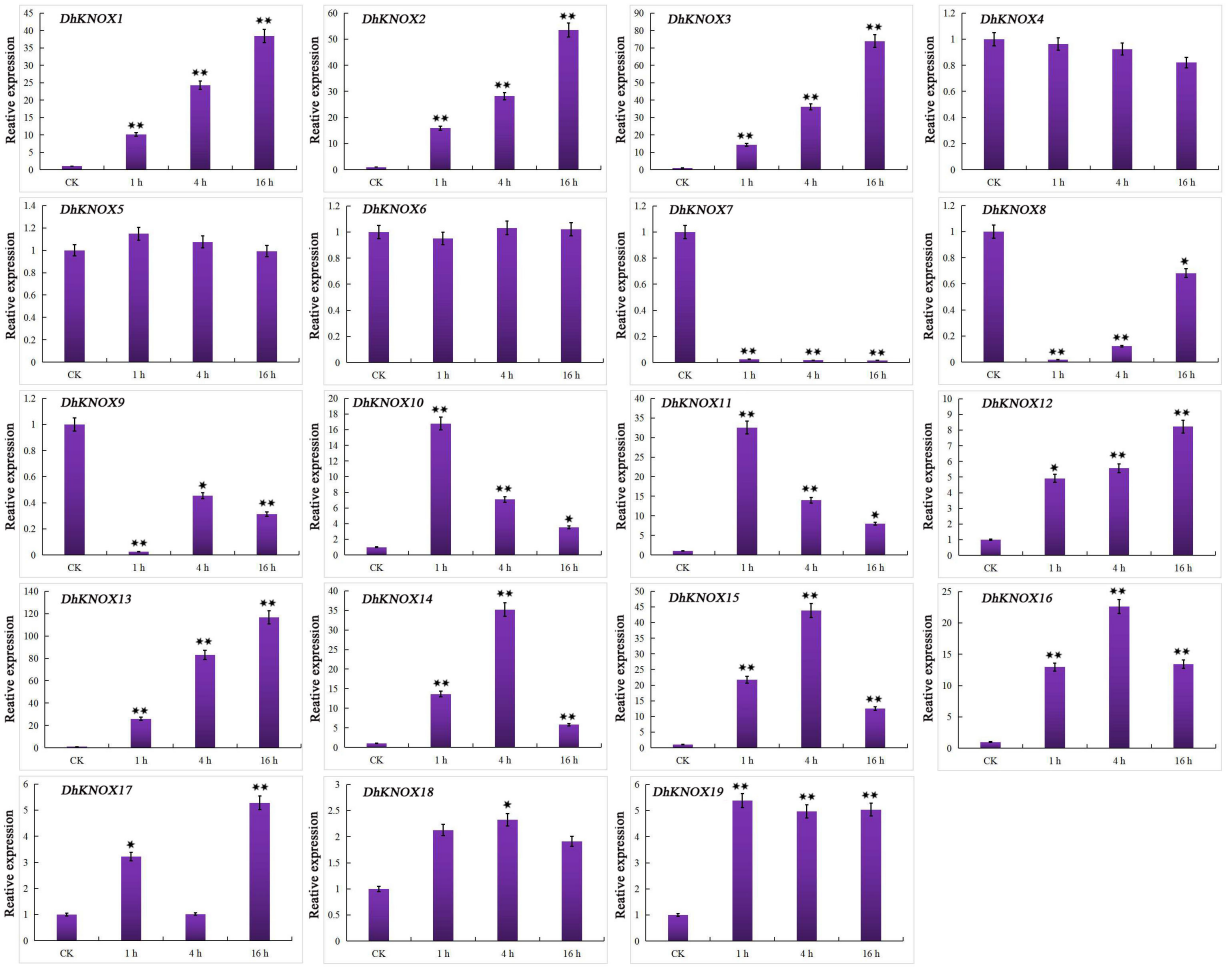
Expression patterns of *DhKNOX* genes in response to PEG treatments. The *x*-axis indicated the time course of each stress treatment, and the *y*-axis represented the relative expression level. The expression data of each gene at 0 h were used as a control sample to show the relative expression level. The data are presented as the mean values ± standard deviation of three biological replicates. Significant differences were determined by ANOVA combined with a *post-hoc* test (Student’s *t*-test). An asterisk indicates significant differences (^*^p < 0.05; ^**^p < 0.01).

The expression level of most *DhKNOX* genes showed a significant increase or decrease under 20% PEG treatment, as shown in **Figure 10**. We further found that the expression levels of the five genes were clustered in class I, class II, and class III and gradually increased with the prolongation of treatment time, and the expression level of *DhKNOX13* increased by 120 times compared to the control group, which contains three MBS elements. In addition, the expression levels of four genes, *DhKNOX4*, *DhKNOX5*, *DhKNOX6*, and *DhKNOX18*, did not show significant changes, and this gene promoter does not contain MBS elements. Additionally, the expression of *DhKNOX7*, *DhKNOX8*, and *DhKNOX9* was consistently suppressed under drought treatment. Moreover, three *DhKNOX* genes (*DhKNOX14*, *DhKNOX15*, and *DhKNOX16*) were clustered in class I, and the expression level of these genes first increased and then decreased, reaching a maximum at 12 h. Furthermore, only two *DhKNOX* genes were clustered in class I, and the expression of these two genes varied irregularly.

## Discussion

Transcription factors are unique to plants and serve a critical function in regulating plant growth and development (Huang et al., 2012; Cao et al., 2017). However, limited research has been conducted on the KNOX transcription factor family of *D. huoshanense*. The ongoing enhancement of *Dendrobium* genome data offers an avenue to investigate whether the *KNOX* gene assumes pivotal roles in plant growth, development, and *Dendrobium’s* reaction to diverse environmental stress conditions.

A composite evolutionary tree was constructed by combining 19 DhKNOX proteins in *Dendrobium* with 68 KNOX protein sequences from different species. The class I subfamily can be divided into BP-like, STM-like, and KNAT2/6-like subfamilies; class II can be divided into KNAT3-5-like and KNAT7-like subfamilies. Functional studies showed that BP and STM play regulatory roles in the biosynthesis of lignin and the development of the secondary cell wall (Cheng et al., 2019). In this study, *DhKNOX5*, *DhKNOX7*, and *DhKNOX19* in KNAT7-like in *Dendrobium* may also be involved in regulating the synthesis of secondary walls. Furthermore, *DhKNOX2* and *DhKNOX3* of the 19 *DhKNOX* members in *Dendrobium* and *BP* in *Arabidopsis* converge into the same branch of BP-like evolution, and *DhKNOX3* and *DhKNOX4* converge into the same group with STM. We hypothesized that two genes belonging to these two subfamilies are involved in the regulation of lignin synthesis.

Considering the functional significance of conserved domains within the KNOX protein, we focused on investigating the KNOX protein domains in *Dendrobium*. We discovered that most members of the same subgroup have at least one KNOX protein with the same motifs, such as DhKNOX6/DhKNOX12 and DhKNOX18/DhKNOX19. In addition, 19 *DhKNOX* genes contain different numbers of exons or introns, which indicates that the *KNOX* gene family of *Dendrobium* has great diversity. Furthermore, research has found that the gene intron can usually be well preserved during evolution (Wang et al., 2015). This may explain the functional differences and diversity of closely related *DhKNOX* genes, such as *DhKNOX2* and *DhKNOX3*, as well as *DhKNOX7* and *DhKNOX 8* (**Figure 4C**).

It has been observed that the regulation of gene expression levels involves the coordination of various *cis*-acting elements (Cao et al., 2018; Soliman and Meyer, 2019). In our research, the DhKNOX promoter also contains hormone response elements, including the CGTCA motif and ABRE motif. Therefore, the *DhKNOX* family may be regulated by these plant hormones or even by hormone-mediated growth and development or stress regulation (**Figure 5**). Furthermore, the study of the gene function of this family in different plants also corroborated the results of the existence of many hormone-related elements in the *cis-*element of the promoter. For example, the *KNOX* gene in *P. paten* can promote cytokinin biosynthesis through the isopentenyl transferase gene PpIPT3 (Coudert et al., 2019) and LcKNAT1 in *Litchi chinensis* can regulate exfoliation by regulating ethylene biosynthesis (Zhao et al., 2020) and participate in the regulation of gibberellin function (Sakamoto et al., 2001; Hay et al., 2002).

In plants, genes that are commonly linked to hormone stress are responsible for generating stress responses. These responses are regulated and/or mediated by diverse hormone signaling pathways (Walther et al., 2007; Cao et al., 2019). For example, the promoter regions of the *KNOX* gene in *Cymbidium* and *Punica granatum* contain abundant MeJA and ABA-responsive *cis*-elements, as well as auxin and gibberellin components, respectively (Wang Y. Y. et al., 2020; Zhang Q. P. et al., 2021), and the expression level of the *KNOX* gene can be influenced by changes in exogenous 6-BA, IAA, and KT signals in *Gossypium hirsutum* (Zhang X. H. et al., 2021). In our study, many common *cis*-acting elements, such as MBS, LTR, HSF, ERE, and ABRE, were identified in the promoter region of DhKNOX. These *KNOX* genes contain at least hormone-acting elements, which indicates that the expression level of these genes will change under the action of hormones.

To deeply understand the hormone response mechanism of the *D. huoshanense KNOX* gene, qRT-PCR experiments under different hormone treatments, such as MeJA, ABA, and SA, in leaves (**Figures 6****–** **8**). The *DhKNOX* gene then showed significant differential expression patterns under treatment with these three hormones. These treatments strongly upregulated some *DhKNOX* genes, indicating that these genes may play a key role in the hormone response of *D. huoshanense*. For example, overexpression of *MdKNOX19* in apples increases the sensitivity of apple callus to ABA (Jia et al., 2021). While eight *DhKNOX* genes presented reduced expression levels to different degrees under MeJA treatment (**Figure 6**). Similar findings have been observed in other plants. For instance, *DhKNOX2* and *DhKNOX3* exhibited notably elevated expression levels under ABA (more than 100 and 500 times, respectively, the CK level) (**Figure 7**). Similarly, *DhKNOX9* and *DhKNOX10* demonstrated substantial upregulation under SA, exceeding 100 and 400 times, respectively, compared to the control (**Figure 8**). These three genes may be more sensitive to ABA and SA hormones of *D. huoshanense* plant growth and development. Among these *DhKNOX* genes, a majority possess conserved domains, and it is worth noting that all *DhKNOX* genes demonstrated hormone responsiveness regardless of the presence of conserved domains. These results suggested that gene-coding proteins with KNOXI, KNOXII, ELK, or HOM domains may play a role in the stress response. In addition, we found that the *DhKNOX* gene is sensitive to different hormones to varying degrees. These results provide evidence that KNOX members can participate in the response to abiotic stress. We conjecture that these three hormones may directly or indirectly regulate the transcription level of *DhKNOX*. In the future, we want to explore the influence of exogenous hormones on the regulation of *DhKNOX* expression level in order to determine whether the growth and development of *Dendrobium* can be altered by regulating exogenous hormones.

In summary, there is a certain correlation between hormone response and plant resistance to abiotic stress. For example, gene expression patterns related to ethylene suggest that ethylene may indirectly participate in the induction of dormant genes, thereby enhancing the cold resistance of *P. mume* (Li et al., 2021). This phenomenon is also found in the TALE gene expression mode, where expression is not only regulated by some hormones but is also sometimes affected by some abiotic stress (Tsuda and Hake, 2015; Niu and Fu, 2022). The expression of the pear *KNOX* gene family can regulate drought stress, especially the transcription level of *PbKNOX7/13*, which is significantly increased under drought stress, while the transcription level of *PbKNOX5/16* is significantly reduced under drought stress (Liu et al., 2022). Notably, the expression of *DhKNOX7*, *DhKNOX8*, and *DhKNOX9* remained consistently suppressed during drought treatment. This observed reduction in transcription levels could potentially contribute to enhancing the resistance of *D. huoshanense* to drought stress. However, a comprehensive and systematic investigation is warranted to fully comprehend the implications of these downregulated gene expressions in bolstering *D. huoshanense’s* resilience.

## Conclusions

In our study, we identified and characterized 19 *DhKNOX* genes from the *Dendrobium* genome. These genes were classified into three major families based on their sequence similarities and phylogenetic relationships. Through promoter analysis, we discovered that several *cis*-acting elements were associated with phytohormone signaling, growth, and development, as well as stress responses in the *DhKNOX* gene family. Moreover, we conducted a comprehensive analysis of the expression patterns of these 19 *DhKNOX* genes under various hormonal treatments, including SA, ABA, MeJA, and drought. Our findings shed light on the important role of *DhKNOX* genes in regulating morphogenesis, growth, and development, particularly in response to abiotic stress conditions. This study serves as a foundation for further investigations into the precise mechanisms and functions of *KNOX* genes in the growth, development, and morphogenesis of *Dendrobium*.

## Data availability statement

The original contributions presented in the study are included in the article/**Supplementary Files**, further inquiries can be directed to the corresponding author/s.

## Author contributions

GL: Data curation, Formal Analysis, Funding acquisition, Investigation, Methodology, Software, Validation, Visualization, Writing – original draft, Writing – review & editing. MM: Formal Analysis, Software, Writing – review & editing. GW: Formal Analysis, Writing – original draft. CC: Conceptualization, Funding acquisition, Project administration, Resources, Supervision, Writing – original draft. CS: Conceptualization, Data curation, Funding acquisition, Project administration, Resources, Software, Supervision, Writing – original draft, Writing – review & editing.
