# Supplementary material for: Comparative analysis of KNOX genes and their expression patterns under various treatments in Dendrobium huoshanense
Source: Front Plant Sci. 2023 Oct 4;14:1258533. doi: 10.3389/fpls.2023.1258533 (PMC10582715; doi:10.3389/fpls.2023.1258533)
Supplement: Supplementary file 1 [file Table_1.docx]

**Table S1** GenBank accession codes used for constructing phylogenetic trees

| **Gene name** | **Accession number** | **Species name** |
| --- | --- | --- |
| *AtKNT1* | AT4G08150 | *Arabidopsis thaliana* |
| *AtKNAT2* | At1g70510 | *Arabidopsis thaliana* |
| *AtKNAT3* | At5g25220 | *Arabidopsis thaliana* |
| *AtKNAT4* | At5g11060 | *Arabidopsis thaliana* |
| *AtKNAT5* | At4g32040 | *Arabidopsis thaliana* |
| *AtKNAT6* | At1g23380 | *Arabidopsis thaliana* |
| *AtKNAT7* | At1g62990 | *Arabidopsis thaliana* |
| *AtSTM* | At1g62360 | *Arabidopsis thaliana* |
| *AtKNATM* | At1g146760 | *Arabidopsis thaliana* |
| *ARK1* | AY755413 | *Populus* |
| *ARK2* | Potri.002G113300.1 | *Populus* |
| *OSH1* | Os03g51690 | *Oryza sativa* |
| *OSH10* | Os03g47016 | *Oryza sativa* |
| *OSH15* | Os07g03770 | *Oryza sativa* |
| *OSH3* | Os03g51710 | *Oryza sativa* |
| *HOS58* | LOC_Os02g08544.1 | *Oryza sativa* |
| *HOS59* | LOC_Os06g43860.1 | *Oryza sativa* |
| *HOS66* | LOC_Os03g03164.2 | *Oryza sativa* |
| *LePTS* | ACA61779 | *Solanum lycopersicum* |
| *LeT6* | AAC49917 | *Solanum lycopersicum* |
| *LeT12* | AAC49918 | *Solanum lycopersicum* |
| *LeTKn1* | AAC49251 | *Solanum lycopersicum* |
| *LetKn2* | AAD00251 | *Solanum lycopersicum* |
| *LeTKn3* | AAD00252 | *Solanum lycopersicum* |
| *LeTKn4* | AAO33774 | *Solanum lycopersicum* |
| *MtKNOX1* | ABO33478 | *Medicago truncatula* |
| *MtKNOX4* | ABO33481 | *Medicago truncatula* |
| *MtKNOX5* | ABO33482 | *Medicago truncatula* |
| *MtKNOX6* | ABO33483 | *Medicago truncatula* |
| *NtH1* | AAO11694 | *Nitotiana tabacum* |
| *NtH9* | BAA76903 | *Nitotiana tabacum* |
| *NtH15* | BAA25546 | *Nitotiana tabacum* |
| *NtH20* | BAA76904 | *Nitotiana tabacum* |
| *NtH201* | BAF95776 | *Nitotiana tabacum* |
| *NtH22* | BAA76905 | *Nitotiana tabacum* |
| *NtH23* | BAA25921 | *Nitotiana tabacum* |
| *NtKn1* | AF544052 | *Nitotiana tabacum* |
| *PpKNOPE1* | ABD52723 | *Prunus persica* |
| *PpKNOPE2* | ABO28750 | *Prunus persica* |
| *PpKNOPE2.1* | JQ038131 | *Prunus persica* |
| *PpKNOPE3* | ACJ71731 | *Prunus persica* |
| *PpKNOPE4* | ABO26062 | *Prunus persica* |
| *PpKNOPE6* | ADC35598 | *Prunus persica* |
| *PpKNOPE7* | JQ038132 | *Prunus persica* |
| *PpKNOPEM* | JQ038133 | *Prunus persica* |
| *PpSTMlike1* | ADC35599 | *Prunus persica* |
| *PpSTMlike2* | ADC35600 | *Prunus persica* |
| *ZmRS1* | NP_001149651 | *Zea mays* |
| *Zmkn1* | NP_001105436 | *Zea mays* |
|  |  |  |
