# Supplementary material for: Comparative analysis of KNOX genes and their expression patterns under various treatments in Dendrobium huoshanense
Source: Front Plant Sci. 2023 Oct 4;14:1258533. doi: 10.3389/fpls.2023.1258533 (PMC10582715; doi:10.3389/fpls.2023.1258533)
Supplement: Supplementary file 2 [file Table_2.doc]

**Table S2 Primer sequences used for qRT-PCR**

| Gene name | Upstream primer （5′→3′） | Downstream primers （5′→3′） |
| --- | --- | --- |
| *DhKNOX1* | TGGGCGCTCCACCGGAGGT | TTGCTGAGTCCACAGGCCTTGAT |
| *DhKNOX2* | GGAATTACACTACAAATGGC | GGAGGCCAGTTCTTAGCTG |
| *DhKNOX3* | AGTTTAGGAGAAACCGTTC | AGGTGGCATTGGCGGAA |
| *DhKNOX4* | TAGCGGTTATTTGAGTAG | TCAAAAGCAAATAAACA |
| *DhKNOX5* | GAACAGTCATTACAAATGGC | ACATTGGAAGCCCTCCGA |
| *DhKNOX6* | TCCCAACCGCTGACCCTGAG | TCGACGTCTTCCTTCCGTCGG |
| *DhKNOX7* | AATTGTGGACATTAG  AGAGG | AGAGAAAGAGAAACTGGCATAGC |
| *DhKNOX8* | CGCCGGAAGATAGTTACCAGAAG | TGGGAATAAGAGAAAGGTTTCTT |
| *DhJKNOX9* | AGCGGAGGCTGAGGTTTCAGCG | ATTCTCCGAGCTTGGCTCTTT |
| *DhKNOX10* | GAGCTGGAGCTGGAGCCTAACAAT | TTGCTAACCTTAGGGAATCCAT |
| *DhKNOX11* | CAATGGATGATTCCAGTTCTTCT | CAAAACTCCCTCTCATCTATCA |
| *DhKNOX12* | TGGCTGCGAAGGAGAGATC | AAGTACTGGCAGATACTGTGG |
| *DhKNOX13* | ATTGTAGGTGTTGAAGGCGG | TGTCCAGGAACCAGGTTTCAAATT |
| *DhKNOX14* | GCAGGATGAGTCAAGCGCTTCT | TAACCTTAGCTCTTCTTCAG |
| *DhKNOX15* | CCTGTTCATGACGAGGAACAG | TCTCAAGGAGCCAGGTCTCCA |
| *DhKNOX16* | CCTGTTCATGACGAGGAACA | GAACAGAGAACTTCGGCATTG |
| *DhKNOX17* | AGCTGCTCAACGAGTTCTGCAA | GAGAACAGAGCATTTCGGTG |
| *DhKNOX18* | GTCCATGTCCAGTGGGCAAG | CTTCCTCAGAAAATGCAACCAA |
| *DhKNOX19* | ACAAACACGATGAGCACAGTT | CGCACAGGAAGACCAACAAAG |
| *Tublin* | AGAACAAGAACTCGTCCTAC | GAACTGCTCGCTCACTCTCC |
